# The piperazine compound ASP activates an auxin response in *Arabidopsis thaliana*

**DOI:** 10.1186/s12864-020-07203-8

**Published:** 2020-11-11

**Authors:** Fengyang Xu, Shuqi Xue, Limeng Deng, Sufen Zhang, Yaxuan Li, Xin Zhao

**Affiliations:** College of Life Sciences, Capital Normal University, Beijing, 100048 China

**Keywords:** Chemical genetics, Auxin response, ASP, Phytohormone, Auxin signaling

## Abstract

**Background:**

Auxins play key roles in the phytohormone network. Early auxin response genes in the *AUX/IAA, SAUR,* and *GH3* families show functional redundancy, which makes it very difficult to study the functions of individual genes based on gene knockout analysis or transgenic technology. As an alternative, chemical genetics provides a powerful approach that can be used to address questions relating to plant hormones.

**Results:**

By screening a small-molecule chemical library of compounds that can induce abnormal seedling and vein development, we identified and characterized a piperazine compound 1-[(4-bromophenoxy) acetyl]-4-[(4-fluorophenyl) sulfonyl] piperazine (ASP). The *Arabidopsis DR5::GFP* line was used to assess if the effects mentioned were correlated with the auxin response, and we accordingly verified that ASP altered the auxin-related pathway. Subsequently, we examined the regulatory roles of ASP in hypocotyl and root development, auxin distribution, and changes in gene expression. Following ASP treatment, we detected hypocotyl elongation concomitant with enhanced cell elongation. Furthermore, seedlings showed retarded primary root growth, reduced gravitropism and increased root hair development. These phenotypes were associated with an increased induction of *DR5::GUS* expression in the root/stem transition zone and root tips. Auxin-related mutants including *tir1–1*, *aux1–7* and *axr2–1* showed phenotypes with different root-development pattern from that of the wild type (Col-0), and were insensitive to ASP. Confocal images of propidium iodide (PI)-stained root tip cells showed no detectable damage by ASP. Furthermore, RT-qPCR analyses of two other genes, namely, Ethylene Response Factor (*ERF115*) and Mediator 18 (*MED18*), which are related to cell regeneration and damage, indicated that the ASP inhibitory effect on root growth was not attributable to toxicity. RT-qPCR analysis provided further evidence that ASP induced the expression of early auxin-response-related genes.

**Conclusions:**

ASP altered the auxin response pathway and regulated *Arabidopsis* growth and development. These results provide a basis for dissecting specific molecular components involved in auxin-regulated developmental processes and offer new opportunities to discover novel molecular players involved in the auxin response.

**Supplementary Information:**

The online version contains supplementary material available at 10.1186/s12864-020-07203-8.

## Background

Auxins are a class of phytohormones that regulate almost every aspect of plant growth and development, including root development, vascular tissue differentiation and tropisms [1, 2]. Indole-3-acetic acid is the best known auxin, and its receptors and signaling pathway are well known [3]. The core components of this pathway are the F-box-containing TRANSPORT INHIBITOR RESISTANT1/AUXIN SIGNALING F-BOX (TIR1/AFB) proteins, the transcriptional corepressors AUXIN/INDOLE-3-ACETIC ACID (Aux/IAA), and the AUXIN RESPONSE FACTOR (ARF) transcription factors. Auxin binds to the receptor TIR1 and members of the AFB family, which are the substrate-recognition subunit of the SUPPRESSOR OF KINETOCHORE PROTEIN 1 (SKP1)/CULLIN1/F-Box (SCF) E3 ubiquitin ligase complex to promote the interaction between TIR1/AFB and Aux/IAA by triggering polyubiquitination and degradation of Aux/IAA. The removal of Aux/IAA allows ARF to activate or repress early auxin-responsive gene transcription [4]. All of these elements contribute to auxin responses.

A growing body of evidence shows that auxin and other phytohormones-signaling pathways can interact with each other; thus, contributing to the complexity of auxin-mediated regulation of plant growth and development [5, 6]. For example, the interaction between auxin and cytokinin pathways has been shown to be important for maintaining plant root and shoot meristematic activity [7]. In this case, hypocotyl cytokinin inhibits auxin and aspartate conjugation, thereby promoting an increase in the concentration of free auxin and, consequently, cytokinin oxidase activity, which in turn leads to cytokinin degradation. In addition, free auxin and auxin conjugates can also inhibit β-glucosidase activity and cause a reduction or loss of cytokinin activity [8]. Considerable attention has also focused on the cross-talk between ethylene and auxin signaling pathways, and it has been unequivocally demonstrated that auxin and ethylene regulate the formation of the apical hook, root, and hypocotyl growth. In *Arabidopsis*, the ethylene mutant *ctr1* showed a short primary root due to inhibited cell division and elongation. This phenotype was associated with an increase in the expression of the auxin transporter *PIN2* and a stronger *DR5::GFP* response [9]. Consistently, in cotton, the effect of ethylene on leaves has been found to be related to a reduction of auxin polar transport [10]. Moreover, auxin can induce ethylene biosynthesis by regulating the expression of 1-aminocyclopropane-1-carboxylic acid (ACC) synthase, a key enzyme in ethylene biosynthesis. In *A. thaliana*, the promoter region of *ACS4* encoding ACC synthase, which contains an auxin response element, can be induced by auxin. In plants, guard cells open and close stomata to control transpiration and regulate gas exchange in leaves. Both cytokinins and auxins have been demonstrated to regulate stomatal behavior [11]. Furthermore, auxin and abscisic acid (ABA) play antagonistic roles in regulating stomatal opening and closure. Such antagonism is achieved by a precise regulation of the ion channel activity of the guard cells, which can reduce IAA or increase ABA, and regulate cytoplasmic pH, thereby affecting the expansion of guard cells [12].

Chemical genetics has been widely used to study biological systems because some small-molecule compounds can specifically bind proteins to interfere with developmental processes [13, 14]. The application of chemical genetics in plants has allowed great progress in identifying plant hormone receptors [15], and signal transduction and plant genetic diversity studies [16].

As important signaling molecules, auxins play a major role in plant growth and development. However, study of the auxin response pathway has been limited to a certain extent by the functional redundancy of early response genes, whereas mutations in key genes can cause plant infertility or lethality. In this study, we examined the effects a small-molecule piperazine compound, ASP, on *A. thaliana* phenotypes, with the aim of identifying and characterizing specific molecular components involved in auxin-regulated developmental processes. Our findings highlight the advantages of using a chemical genetics approach to address questions relating to phytohormone signaling pathways.

## Results

### Screening of small-molecule compounds that alter leaf-vein patterns and the auxin response in *A. thaliana*

**Fig. 1:**
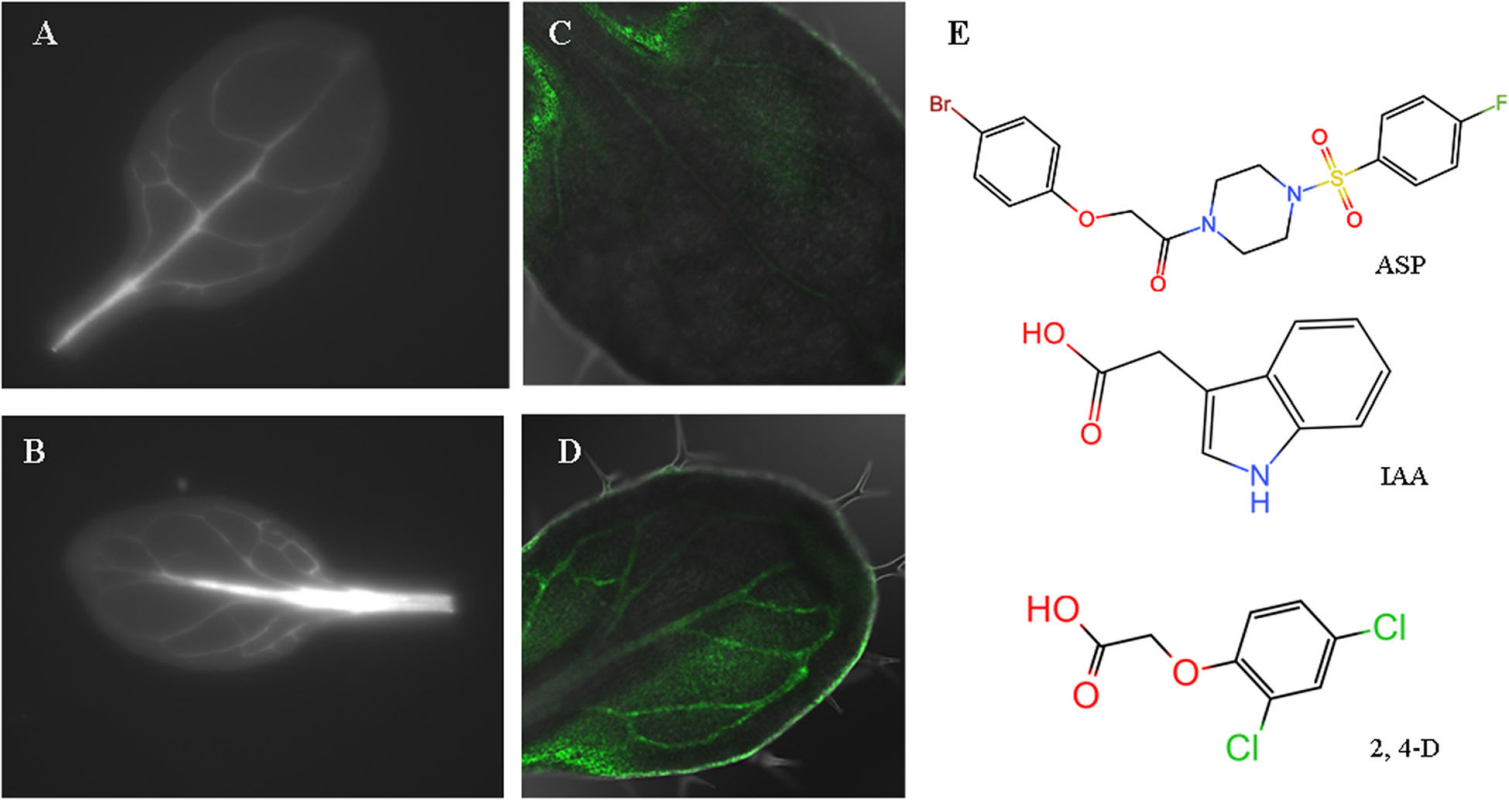
Chemical genetics screening identified a novel compound ASP that produced abnormal leaf-vein pattern. **a-b**: Leaf vein pattern of 10-day-old seedlings of Q0990 line grown on 1/2 MS medium (**a**) and 1/2 MS medium with 10 μM ASP (**b**). **c-d**: Observation of 10-day-old seedlings of *DR5::GFP* reporter line grown on 1/2 MS medium (**c**) and 1/2 MS medium with 10 μM ASP (**d**) by confocal scanning laser microscope. E: Chemical structural formula of endogenous auxin indole-3-acetic acid (IAA), synthetic auxin 2,4-dichlorophenoxyacetic acid (2,4-D), and small-molecule compound 1-[(4-bromophenoxy) acetyl]-4-[(4-fluorophenyl) sulfonyl] piperazine (ASP)

In an attempt to identify small-molecule compounds that can affect leaf vein patterns, we screened a small-molecule compound library containing 3800 compounds, using 10-day-old seedlings of *A. thaliana* line Q0990. The expression of a vascular precursor cell reporter in Q0990 seedlings enabled us to screen for chemically induced defects in vascular development [17]. Seeds of Q0990 were grown in 96-well plates, in which each well contained a 10 μM solution of different compounds. Among these compounds, we selected 11 small molecules having an effect on vein pattern and leaf shape, all of which promoted central-vein thickening and an increase in the number of parallel vascular bundles (Fig. 1a, b).

To determine whether these physiological effects were correlated with a molecular auxin response, we employed transgenic *Arabidopsis* seedlings in which the synthetic auxin response element DR5 was fused to the green fluorescent protein (GFP) reporter. Among the 11 initially selected chemicals, we identified six candidates by confocal microscopy, in which the expression level of the reporter gene increased significantly after treatment, and the distribution of which was consistent with the location of leaf vein formation (Fig. 1c, d). Here, we focused on analyzing the character of the piperazine compound 1-[(4-bromophenoxy) acetyl]-4-[(4-fluorophenyl) sulfonyl] piperazine (ASP) and used it as a chemical tool to study the auxin signaling pathway.

Notably, we found that ASP showed structural similarities to the synthetic auxin 2,4-dichlorophenoxyacetic acid (2,4-D) and endogenous IAA, both of which contain an unsaturated aromatic ring and a carboxylic acid side chain (Fig. 1e). This structural similarity reminded us of the functional similarity.

### ASP can inhibit the elongation of primary and lateral roots, promote root hair development, and reduce gravitropism in *A. thaliana*

**Fig. 2:**
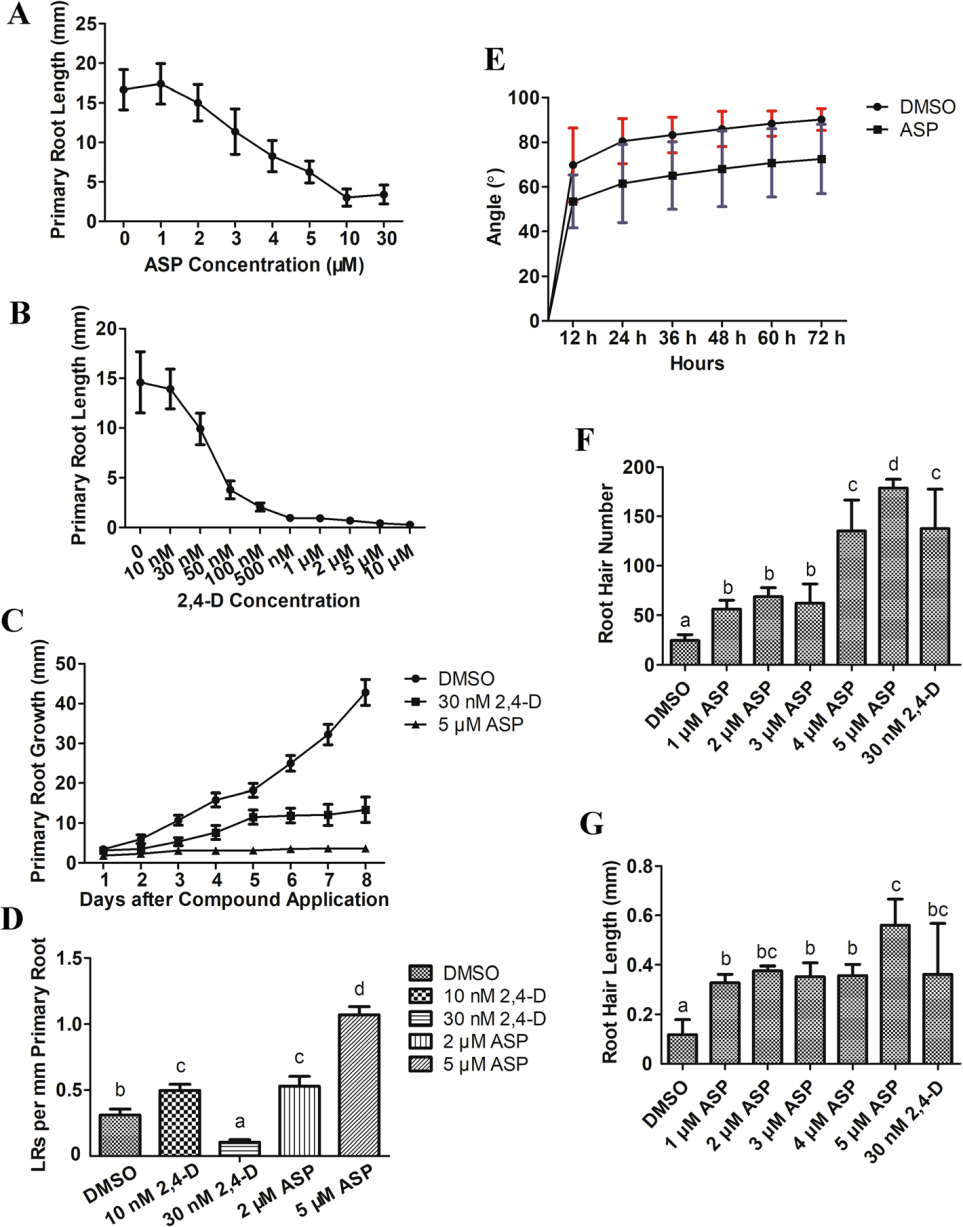
ASP decreased root growth in *A. thaliana*. **a**: Dose-response curve for wild type (Col-0) seedlings on ASP. Seedlings were treated for six days with different concentrations of ASP from 0 to 30 μM as indicated on X axis. **b**: Dose-response curve for Col-0 seedlings on 2,4-D. The concentrations of 2,4-D were from 10 nM to 10 μM as indicated on X axis. **c**: The cumulative rate of primary root length measured at 24-h interval. Seedlings were treated with 5 μM ASP and 30 nM 2,4-D respectively. DMSO was used as control. Each point represented the mean of at least 20 measurements. Error bars indicated the standard deviation. **d**: Effect of ASP and 2,4-D on lateral root density of WT seedlings. Seedlings were grown on 1/2 MS medium supplemented with 10 nM 2,4-D, 30 nM 2,4-D, 2 μM ASP and 5 μM ASP for nine days. DMSO was used as control (number of samples *N* = 10, *P* < 0.05). **e**: ASP reduced the gravitropic response. 2 μM ASP was used for gravitropism test. Col-0 seedlings grown on agar medium were rotated 90° at time 0. The angle of curvature from the horizontal was measured at the times indicated. Each point represents the mean of 24 measurements. Error bars indicate the standard deviation. **f** and **g**: Root hair of Col-0 seedlings response to ASP and 2,4-D. Seedlings vertically grown on the medium supplemented with 0–5 μM ASP and 30 nM 2,4-D for six days. Root hair upward root tips 5 mm was selected to measure and count (number of samples *N* = 50, *P* < 0.05). **f**: Statistics of root hair number. **g**: Measurements of root hair length

Phytohormones such as auxin and ethylene play crucial roles in the regulation of root growth [18]. To further characterize ASP activity, we accordingly performed a series of root assays. Following treatment of 6-day-old seedlings of wild-type *A. thaliana* (Col-0) with different concentrations of ASP and 2,4-D, we found that both compounds had clear concentration-dependent inhibitory effects on root growth (Fig. 2a, b) (Additional file 1: Fig. S1). When we measured primary root length at 24-h intervals, we found that the rate of primary root cumulative growth was reduced in the 5 μM ASP and 30 nM 2,4-D treatments (Fig. 2c). Furthermore, we observed that 30 nM 2,4-D and 4 μM ASP had similar effects on primary root growth (Fig. 2a, b) (Additional file 1: Fig. S1), and that under these treatments, the inhibition rate of primary root growth was 50%. Therefore, in subsequent experiments, 30 nM 2,4-D was used as positive control treatment.

Then we observed the lateral root growth in 9-day-old seedlings, we found that 10 nM 2,4-D increased the lateral root density, however, 30 nM 2,4-D inhibited such roots formation (Fig. 2d). When seedlings were treated with 2 μM and 5 μM ASP, we observed the emergence of numerous lateral roots on the primary root, although lateral root elongation was inhibited (Additional file 2: Fig. S2). Given that the effect of 10 nM 2,4-D and 2 μM ASP had similar effects on the lateral root density of WT, we speculated that although ASP promoted lateral root initiation, it inhibited lateral root elongation.

Gravitropism is the process whereby plants orientate their root growth toward gravity, and is a necessary response that ensures roots grow down through the soil [19]. We examined whether ASP had an effect on the gravitropic response of Col-0 seedlings by rotating seedlings grown vertically on 2 μM ASP-containing medium, and accordingly found that ASP retarded the rate of the gravitropic response. Thus, at 12 h after treatment, reorientation of the roots of ASP-treated plants showed noticeable inhibition, with a growth angle of 53° compared with the 70° of control plant roots. This trend became increasing more pronounced with continued root growth, and when final measurements were taken at 72 h after treatment, we found that the primary roots of ASP-treated plants had not bent to an angle of 90° (Fig. 2e, Additional file 3: Fig. S3).

Root hair development has been shown to be regulated by multiple plant hormones [20], and in the present study, we observed an increase in the number of root hairs growing on Col-0 seedlings in response to 1–5 μM ASP treatment; further, the length of these root hairs changed significantly (Fig. 2f, g). Moreover, we observed that the growth of root hairs occurred closer to the root tips (Additional file 4: Fig. S4). These findings thus indicated that ASP can inhibit primary and lateral root growth, retard the gravitropic response, and promote root hair production and growth in WT *Arabidopsis* seedlings.

Based on the effect of ASP on root growth, we suspected that root inhibition was due to the toxicity of ASP. We tested this possibility by using propidium iodide (PI)-stained root tip cells of 5-day-old seedlings treated with different concentrations of ASP, IAA and 2,4-D. PI is a nucleic-acid stain that can only penetrate cells with damaged or leaking cell membranes [21]. Confocal images revealed no detectable cellular damage due to treatment with ASP, IAA or 2,4-D, thereby indicating that the ASP-induced changes in root morphology are not associated with DNA damage or cell death (Additional file 5: Fig. S5).

### ASP promotes hypocotyl elongation in *A. thaliana*

**Fig. 3:**
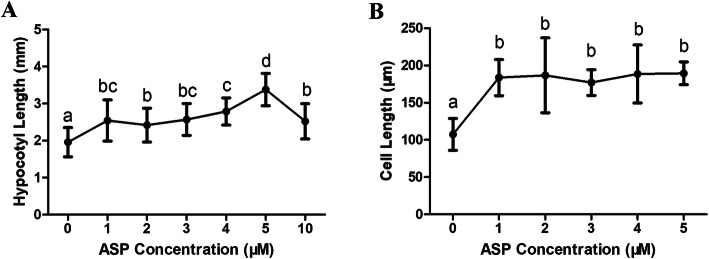
ASP promoted hypocotyl elongation in Col-0 seedlings*.* **a**: Measurements of hypocotyl length in five-day-old seedlings under ASP treatment. The concentrations of ASP application were indicated on X axis. **b**: Statistical results of hypocotyl cell length. Hypocotyl cells were from semi-thin longitudinal section cut (*N* = 60, *P* < 0.05)

In general, well-known auxins (e.g., IAA, 2,4-D, 1-NAA) have no discernable effects on hypocotyl elongation [22]. In the present study, however, we found that treatment of *A. thaliana* with different ASP concentrations promoted hypocotyl elongation; furthermore, it had no clear concentration-dependent effects on 5-day-old seedlings (Fig. 3a, Additional file 1: Fig. S1). We observed semi-thin sections of hypocotyl tissue using light microscopy and measured cell length after 7 days of treatment with ASP to examine its effect on hypocotyl elongation at the cellular level. Longitudinal sections revealed that the hypocotyl cells of ASP-treated plants were significantly longer than those of control plants (Fig. 3b, Additional file 6: Fig. S6). Accordingly, these observations indicated that ASP enhanced hypocotyl growth by promoting cell elongation.

### ASP has differing effects on auxin signaling mutants, and can induce the differential expression of several key genes in the auxin signaling pathway

**Fig. 4:**
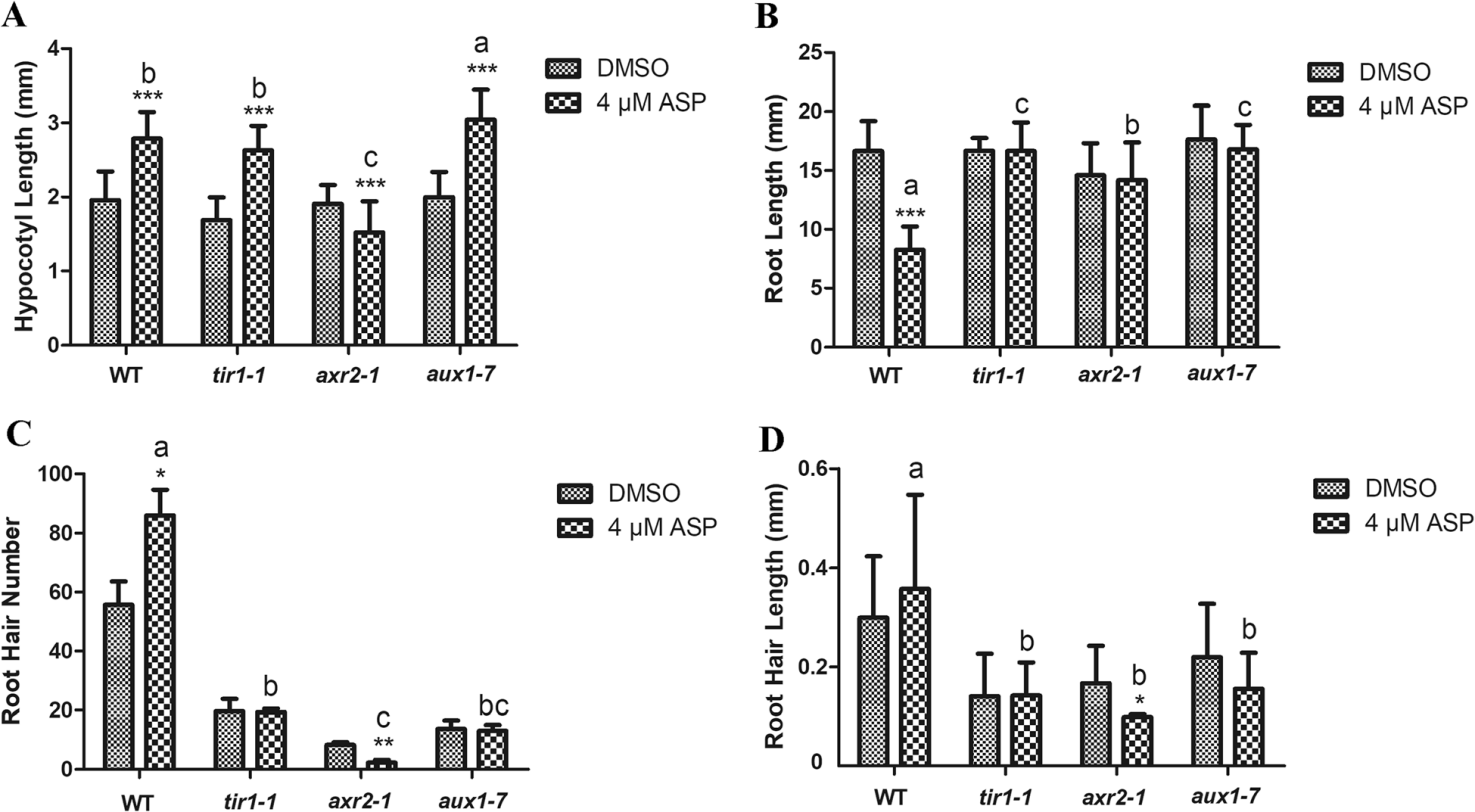
Effects of ASP in *Arabidopsis* auxin signaling mutants. Wild-type and *tir1–1*, *axr2–1* and *aux1–7* mutants were grown on 1/2 MS medium supplemented with 4 μM ASP for six days. DMSO was used as control. **a**: Hypocotyl length. **b**: Primary root length. Data of A and B represent Means ± SD (*N* = 30, *P* < 0.05). **c**: Root hair number. **d**: Root hair length. Data of C and D represent Means ± SD (*N =* 3, *P* < 0.05). The experiment was repeated three times with similar results. * *P* < 0.05, ** *P* < 0.01, *** *P* < 0.001 or different letters indicate means statistically different at *P* < 0.05

To further explore whether ASP acted through the auxin signaling pathway, we assayed the phenotypes of three auxin signaling mutants, namely, *tir1–1*, *axr2–1* and *aux1–7*. Treatment with 1–5 μM ASP increased the hypocotyl length in *tir1–1* and *aux1–7* relative to controls; and all three mutants showed no obvious root growth reduction. Furthermore, the promotion effects on the number and length of root hair were weakened in these mutants (Additional file 7: Fig. S7, Additional file 8: Fig. S8). In treatment with 4 μM ASP that inhibited root elongation rate by 50%, hypocotyl length of WT seedlings actually increased, with the promotion effect in *tir1–1* and *aux1–7* mutants being similar or larger than in the WT (Fig. 4a). However, compared with WT, the inhibitory effect in primary root growth was not significant in *tir1–1*, *axr2–1* and *aux1–7* (Fig. 4b). Meanwhile, the root hair number and length in *tir1–1* and *aux1–7* had no obvious changes by 4 μM ASP treatment, indeed in *axr2–1* they showed significant reduction (Fig. 4c, d). From these results we concluded that the sensitivity of *tir1–1* and *aux1–7* to ASP in the root was different from that in the hypocotyl; therefore, we hypothesized that ASP affected root and hypocotyl growth via different pathways.

**Fig. 5:**
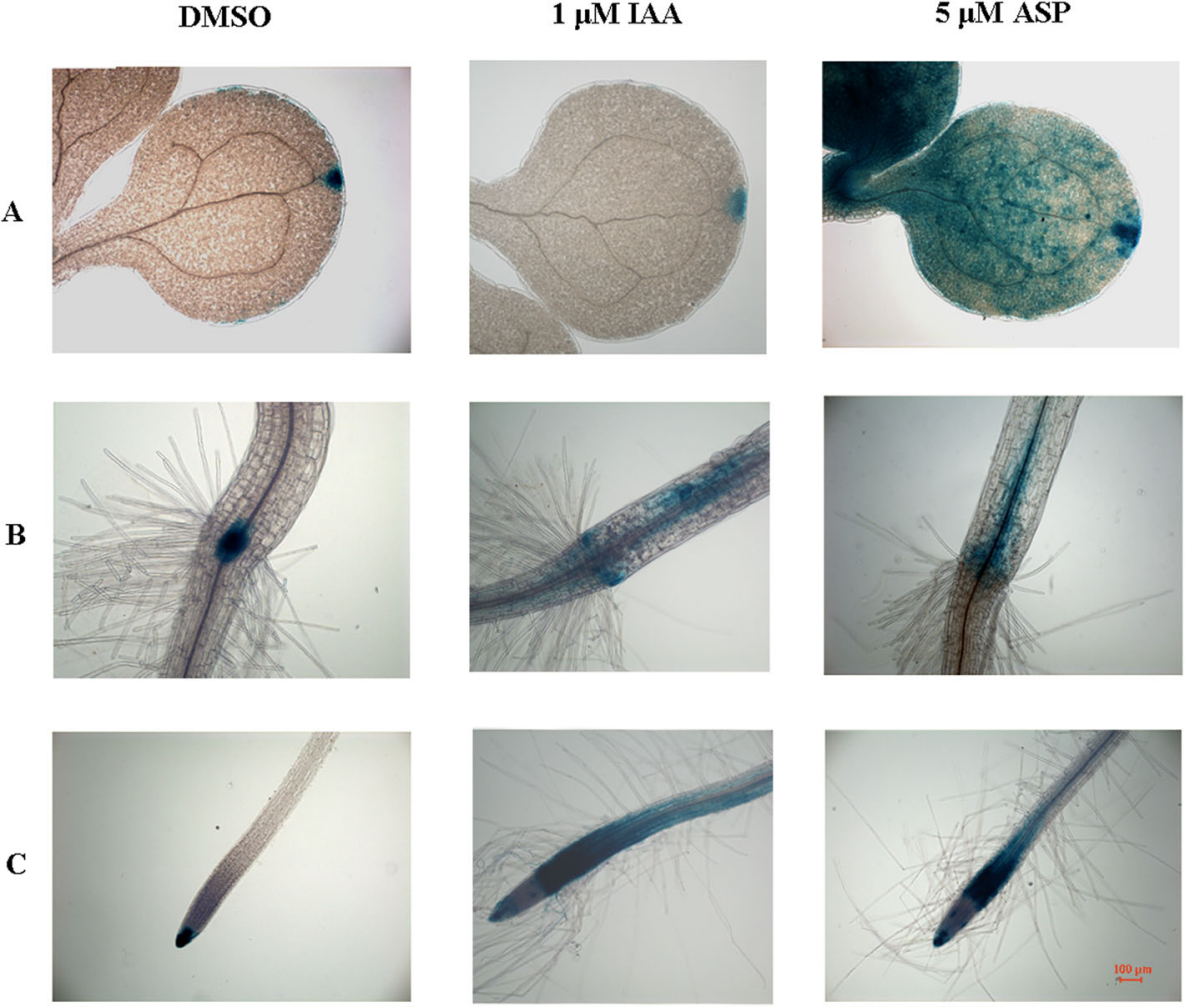
Effect of ASP on auxin response in *Arabidopsis*. Seedlings grown on 1/2 MS agar medium with DMSO for 5 days and then transferred into Eppendorf tube (10 seedlings per tube) containing 1 ml of 1/2 MS liquid medium supplied with DMSO, 1 μM indole-3-acetic acid (IAA) and 5 μM ASP and incubated for 24 h. After ten hours of β-glucuronidase (GUS) staining seedlings were cleared for microscopy analysis. GUS- expressing in cotyledon (**a**), root/ stem transition (**b**), and root tip (**c**) treated for 24 h with DMSO (Left), 1 μM IAA (Middle) and 5 μM ASP (Right). Photographs show representative individuals from at least 20 stained plants (Scale bar = 100 μm)

In addition, we used seedling lines containing the auxin response element DR5 fused to the β-glucuronidase (GUS) reporter to examine whether the effects of ASP were correlated with the auxin response. Histochemical staining of *DR5::GUS* seedlings grown for 24 h in the presence of 5 μM ASP revealed strong GUS expression in the cotyledons (Fig. 5a), the root/stem transition zone (Fig. 5b), and root tips (Fig. 5c). It is well known that auxin inhibits root growth and promotes root hair development [23]; thus, increased auxin accumulation implies root inhibition and root hair promotion.

On the basis of the aforementioned results, we hypothesized that ASP might induce auxin-like responses, and accordingly sought to examine the ability of ASP to induce auxin-response genes in 6-day-old seedlings. To this end, we performed real-time quantitative PCR (RT-qPCR) to analyze the expression of early auxin-response genes including, *Aux/IAA*, *GH3* and *SAUR* within 2 h of treatment with ASP.

**Fig. 6:**
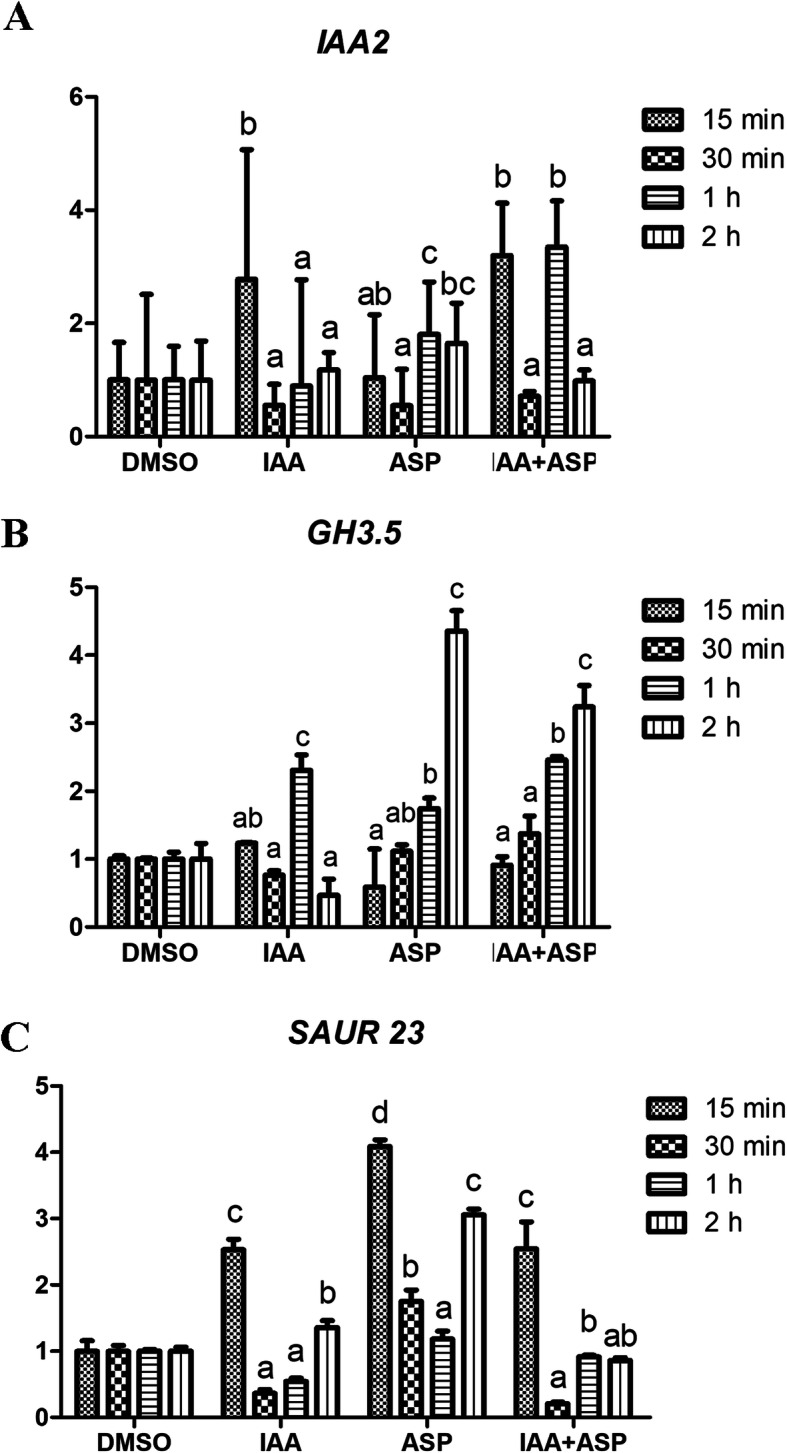
RT-qPCR expression analyses of early auxin response genes. Six-day-old seedlings were treated with 5 μM ASP and 5 μM IAA for 0.5–2 h. Expression level shown are the Means ± SD from three biological replicates for each. Different letters indicate significant differences at *P* < 0.05

As expected, we found that ASP significantly up-regulated the auxin-responsive genes in WT seedlings. *IAA2* expression increased at 15 min by IAA treatment and then decreased over the following 2 h. However, with ASP treatment, the expression level increased at 1 and 2 h sampling-time points. The trend of the relative expression of the *GH3.5* was generally consistent under IAA and ASP treatment. The increase in gene expression level was evident after 1 h treatment, and an over four-fold increase was observed after 2 h of ASP treatment. The response of *SAUR23* to IAA and ASP was rapid. After 15 min of treatment, the expression level had increased significantly (Fig. 6). Although the expression patterns of these genes were not exactly unanimous, the altered expression levels of the examined genes indicated that ASP treatment triggered auxin activity.

## Discussion

Auxins were the first class of plant hormones to be discovered. Auxins play an important role in plant morphogenesis and development, including shoot and root growth and hypocotyl elongation [24]. IAA is the main form of auxin in plants, although there are numerous other substances with auxin-like activity including, indole-3-butanoic acid (IBA), 4-chloroindole-3-acetic acid (4-Cl-IAA), and phenylacetic acid (PAA). These latter compounds have chemical structures similar to that of IAA and, accordingly, are considered to be plant endogenous auxins [25]. Along with the rapid development of synthetic chemical growth regulators, a number of auxin-like compounds have been identified and extensively applied in plant research [26].

Taking advantage of the power of chemical genetics, we performed a small-molecule library screening and obtained six candidates that were considered to be potentially relevant to the auxin response; then, we examined the mode of action of a small-molecule of the piperazine type (designated ASP).

ASP is a piperazine compound with structural similarity to auxins; particularly, to PAA and 2,4-D. All three of these compounds have a benzene ring structure and a carboxyl group (or derivative), which are two common features recognized as critical for auxin activity; however, they differ with respect to the types and sites of the side chains on the benzene ring. In addition, cyclodipeptides and their derivative diketopiperazines (DKPs) synthesized by *Pseudomonas aeruginosa* also possess a heterocyclic system and exhibit auxin-like activity [27]. Both ASP and DKPs belong among piperazines and show similar effects on modulating root architecture and inducing auxin response-related gene expression in *Arabidopsis*, which suggested ASP might be involved in the interaction of signaling-pathways between plants and bacteria.

In the experiments reported herein, we found that ASP effectively promoted hypocotyl elongation, whereas traditional auxins (e.g., IAA, 2,4-D, and NAA) had no effects in this respect under normal light conditions [28]. However, auxin overexpressing mutants, such as *supperroot1* (*sur1*) and *sur2*, were characterized by a long hypocotyl and short roots, a phenotype that can be mimicked by exogenous application of the auxin analog picloram [29]. Nonetheless, it has been shown that the activity of picloram differs from that of 2,4-D with respect to the auxin receptor family member AFB5 [30]. Furthermore, *yucca* mutants were identified with long hypocotyls by activation tagging [31]. In addition to long hypocotyls, *yucca* plants showed phenotypes characteristic of elevated auxin levels during all stages of development; namely, *yucca* seedlings showed epinastic cotyledons and elongated petioles when grown under white light, similar to known auxin overproducing mutants *sur1* and *sur2.* In addition, altered conditions of nutrition, light, and temperature, can potentially contribute to the induction of hypocotyl elongation [32]. The findings of a study on auxin analogs revealed that certain new auxin analogs, acting as “pro-auxins,” can diffuse efficiently to the hypocotyls, wherein they undergone cleavage at differing rates, thereby releasing functional auxins [22]. On the basis of the structure of ASP, we propose that it might also be hydrolyzed to yield an auxin-like active substance which, through tissue-specific localization, gains access to the hypocotyl—previously deemed inaccessible to exogenous auxins. The importance of such possibility warrants further study.

Given that auxins have been shown to inhibit primary root elongation while increasing lateral root development [33], we compared the effect of ASP and 2,4-D on primary root and lateral root growth, gravitropism, and root hair development. We found that primary root growth and gravitropism of WT seedlings was inhibited by ASP; similar to the phenotype induced by treatment with synthetic auxins. Furthermore, ASP induced a root morphology similar to the phenotype of *MED18* loss-of-function mutant. *MED18* is involved in root morphogenesis and is important for cell viability. Mutant *med18* exhibited slow primary root growth and increased root hair formation, and DNA damage occurred in dead cells of root meristems [34]. Meanwhile, *ERF115* (Ethylene Response Factor) is helpful for sustaining meristematic activity via the enhancement of cell replenishment in response to cell damage and *ERF115* expression is induced following cell damage. However, when we tested the expression levels of *MED18* and *ERF115* in the root tips of 6-day-old WT seedlings treated with 2 and 5 μM ASP using RT-qPCR, the expression of these genes was distinct from that in the *med18* mutant. A high expression level of *MED18* and a reduced expression level of *ERF115* resulted from 5 μM ASP treatment (Additional file 9: Fig. S9). We accordingly deduced that the root morphological changes induced by ASP were not due to DNA damage or cell death. We confirmed this conclusion using PI staining: the detection of cell viability in the root tip meristem did not show any change under different concentration of ASP, 5 μM IAA, or 30 nM 2,4-D. Further, we observed that ASP had diverse effects on hypocotyl and root growth in auxin-signaling mutants. The hypocotyl length in *tir1–1* and *aux1–7* increased with 1–5 μM ASP relative to controls. However, the inhibitory effect on root growth had not been observed in these mutants. Accordingly, we suspected that ASP affected root and hypocotyl growth via different pathways.

On the basis of its structure and induced phenotypic characteristics in treated plants, we propose that ASP might be useful as a novel auxin analog. Histochemical staining using *DR5::GUS* confirmed that ASP interferes with the auxin response pathway, and RT-qPCR analysis indicated that early auxin response genes, including those in the *AUX/IAA, GH3* and *SAUR* gene families, were significantly up-regulated via rapid induction by ASP, thereby confirming the auxin-like nature of ASP.

## Conclusions

In this study, we identified the piperazine small-molecule compound ASP based on high-throughput chemical screening. We found that ASP affected the early development of *A. thaliana*, with significant effects on leaf shape and venation pattern. Additionally, ASP had an inhibitory effect on the growth of primary roots but was found to promote hypocotyl elongation and reduced gravitropism in *A. thaliana* seedlings. Moreover, using RT-qPCR, we found that ASP induced the expression of early auxin response genes. Collectively, our findings indicate that ASP can regulate the growth and development of *A. thaliana* by activating an auxin response and inducing the expression of genes involved in the auxin signaling pathway. In addition to demonstrating the effects of the small-molecule compound ASP on *A. thaliana* seedlings at the phenotypic and cellular levels, our studies clearly highlight the advantages of using a chemical genetics approach to dissect the intricacies of plant hormone signaling-pathways.

## Methods

### Plant materials and reagents

*Arabidopsis* Columbia-0 wild type (WT), auxin related mutants such as *tir1–1* (CS3798), *aux1–7* (CS3074) and *axr2–1* (CS3077) seed lines, and *DR5::GFP* and *DR5::GUS* transgenic lines were used for phenotype and mode of action study. These seed lines were preserved in our laboratory. Q0990 line was used to screen chemicals. This line was a kind gift from Prof. Enrico Scarpella, University of Alberta, Canada. Seeds of *A. thaliana* were surface sterilized with 70% ethanol and 10% sodium hypochlorite and dried in sterile bench. The sterilized seeds were germinated on half-strength Murashige and skoog salt (1/2 MS) medium supplemented with 0.1% sucrose and 0.7% plant agar, and grown at 22 °C–24 °C with a light/dark cycle of 16/8 h. All reagents were purchased from Sigma (St. Louis, MO, USA). Stock concentrations of 50 mM ASP and 100 mM 2,4-D were prepared in DMSO. The working concentrations were as follows: 10–100 nM 2,4-D, 0–30 μM ASP.

### Selection of 3800 small-molecule compounds

The Q0990 strain (provided by Enrico Scarpella of University of Alberta) contains the GFP protein and can excite green fluorescence in the vascular system. Screening process: Unbiased small molecule organic compounds were purchased from ChemBridge and stored in 96-well plates, one compound per well. Add one seed per well in a 96-well plate, and then add liquid 1/2 MS broth for a final concentration of 10 μM compound and 0.1% DMSO. The same compound was treated simultaneously in two replicate plates with 1/2 MS + DMSO served as the control. The first pair of true leaves was observed after cold stratification at 4 °C in darkness for 2 days and light culturing at 23 °C (16 h light/8 h dark) for 9 days. These were subsequently analyzed by confocal microscopy (Zeiss live 5).

### Phenotype investigation

The seedlings grew in the chemical medium and the control group was photographed using a Nikon camera. The hypocotyls and root length of each *A. thaliana* seedling were measured using Image J software. At least twenty plants each from the control and treatment groups were analyzed.

### Microscopy observation of hypocotyl cell

The hypocotyls of *A. thaliana* seedlings that had been cultured for 7 days were soaked in immobilized solution and fixed at 4 °C for more than 24 h. Dehydration was carried out at an alcohol gradient of 30 to 50% to 70 to 75% to 80% to about 85% to about 90% to about 100%. The embedding agent was then infiltrated into the material and polymerized at 4 °C for 24 h. The sections were sliced at a thickness of 5 mm and stained to observe the cells under microscopy.

### Growth, curvature, and analysis of Gravitropic sensitivity

Wild-type *A. thaliana* seeds were cultured on 1/2 MS medium for 4 days. Uniform seedlings were then selected and vertically grown in 2 μM-ASP medium for 24 h. The plates were rotated clockwise 90° to make the root parallel to the ground. We observed and photographed the root every 12 h. Image J software was used to measure the curvature of 20 plants each in the control and treatment groups.

### Histochemical analysis

Five-day-old Arabidopsis seedlings grown on MS medium with or without 5 μM ASP were incubated at 37 °C in a GUS staining solution (O’BioLab Co Ltd., Beijing, China) for 10 h. Chlorophyll was removed by washing plants several times with 70% (v/v) ethanol, and then observed under stereoscopic microscope zeiss, Axio Zoom.V16, Germany.

### RNA isolation and quantitative Reverese-transcription polymerase chain reaction

Total RNA was isolated using TRIzol reagent (Invitrogen) according to manufacturer’s instructions. First-strand cDNA was synthesized with 5xPrimeScript RT Master Mix (Takara). RT-qPCR was performed in the ABI (Quant studio 6, USA) using the 2 × SYBT Premix Ex TaqTM (Takara) and analysed using “Quant studio-Real-Time PCR” software. Primers are described in the Additional file 10: Table S1.

### Propidium iodide staining

For fluorescence staining with PI, plants were transferred from the growth medium to 10 mg/mL PI solution for 2 min. Seedlings were rinsed in phosphate-buffered saline (PBS) and mounted in water on microscope slides. Samples were observed under a laser-scanning confocal microscope (LSCM: Zeiss 780; Germany) at wavelengths specific to PI fluorescence using a 561-nm excitation line [34].

### Statistical analysis

For all experiments the overall data were statistically analyses in the spss19.0 software. All results were presented as the mean ± SD. using GraphPad Prism 5.00 (GraphPad Software, San Diego, CA, USA) and analyzed using Student’s t test or ANOVA (analysis of variance). Univariate analyses with a Tukey’s-b (k)/Duncan (D) post hoc test were used for testing differences. Different letters are used to indicate means that differ significantly (*P* < 0.05).

## Supplementary Information

**Additional file 1: Fig. S1** Representative images of *Arabidopsis* WT (Col-0) seedlings grown on petri plates with medium supplemented with 2,4-D or ASP. DMSO was used as control. Concentrations are indicated under each image (Scale bar =15 mm).

**Additional file 2: Fig. S2** Representative images of *Arabidopsis* WT (Col-0) seedlings supplemented with 2,4-D or ASP. Seedlings were grown on 1/2 MS liquid medium for nine days supplied with different concentrations of ASP or 2,4-D. DMSO was used as control. Concentrations are indicated under each image (Scale bar =15 mm).

**Additional file 3: Fig. S3** Testing gravitropism of rotated roots with 2 μM ASP treatment for 12–72 h.

**Additional file 4: Fig. S4** Representative images showing the root-hair phenotypes of *Arabidopsis* WT (Col-0) seedlings. Seedlings were grown on ASP or 2,4-D-supplemented medium for 6 days. DMSO was used as control. Concentrations are indicated on each image (Scale bar = 500 μm).

**Additional file 5: Fig. S5** Images of propidium iodide (PI)-stained root tip cells. Primary root tips from Col-0 seedlings, which grown on 1/2 MS medium with 0–5 μM ASP, 5 μM IAA and 30 nM 2,4-D for five days, stained with 10 mg/ml PI for 2 min. Images were acquired using a Zeiss LSCM 780 confocal microscope (Scale bar = 50 μm).

**Additional file 6: Fig. S6** ASP promoted hypocotyl cell elongation. Semi-thin longitudinal section cut from hypocotyl tissue cells and observed with light microscopy (Scale bar = 50 μm).

**Additional file 7: Fig. S7** Comparison of auxin signaling mutants (*tir1–1*, *axr2–1*, *aux1–7*) with wild-type seedlings on hypocotyl and root growth. The seedlings were grown on medium supplemented with 0–5 μM ASP for six days. A: Hypocotyl length. B: Root length. Means ± SD values were shown, *N* = 30 seedlings. C: Root hair number. D: Root hair length. Means ± SD values were shown, *N* = 10 seedlings. Micromolar concentrations are indicated on X axis.

**Additional file 8: Fig. S8**: Representative root hair phenotypes of auxin-related mutants. A: Wild-type and mutant (*tir1–1*, *axr2–1*, *aux1–7*) seedlings were grown on 1/2 MS liquid medium for 6 days. B: Wild-type and mutants were treated with 4 μM ASP for 6 days (Scale bar = 500 μm).

**Additional file 9: Fig. S9**: RT-qPCR expression analyses of *ERF115* and *MED18* to test the root meristem cell viability. The seedlings were grown on 1/2 MS medium supplemented with 2 and 5 μM ASP for six days. DMSO was used as control.

**Additional file 10: Table S1** Sequences of primers used in RT-qPCR.

## Data Availability

All data generated or analysed during this study are included in this published article and its supplementary information files. IAA2 (AT3G23030), GH3.5 (AT4G27260), SAUR23 (AT5G18060), PIN2 (AT5G57090), ERF115 (AT5G07310), MED18 (AT2G22370).

## Abbreviations

2,4-D: 2,4-Dichlorophenoxyacetic acid
4-Cl-IAA: 4-Chloroindole-3-acetic acid
*A. thaliana*: *Arabidopsis thaliana*
ABA: Abscisic acid
ACC: 1-Aminocy-clopropane-1-carboxylic acid
ARF: Auxin response factor
ASP: 1-[(4-Bromophenoxy) acetyl]-4-[(4-fluorophenyl) sulfonyl] piperazine
Aux/IAA: Auxin/Indole-3-acetic acid
DMSO: Dimethyl sulfoxide
HSD: Honestly significant difference
IAA: Indoleacetic acid
IBA: Indole-3-butanoic acid
JA: Jasmonic acid
NAA: 1-Naphthylacetic acid
PAA: Phenylacetic acid
SA: Salicylic acid
SKP1: Suppressor of kinetochore protein 1
WT: Wide type
PI: Propidium iodide
PBS: Phosphate-buffered saline
LSCM: Laser scanning confocal microscope
