# Supplementary figures and images for: The piperazine compound ASP activates an auxin response in Arabidopsis thaliana
Source: BMC Genomics. 2020 Nov 11;21:788. doi: 10.1186/s12864-020-07203-8 (PMC7659159; doi:10.1186/s12864-020-07203-8)

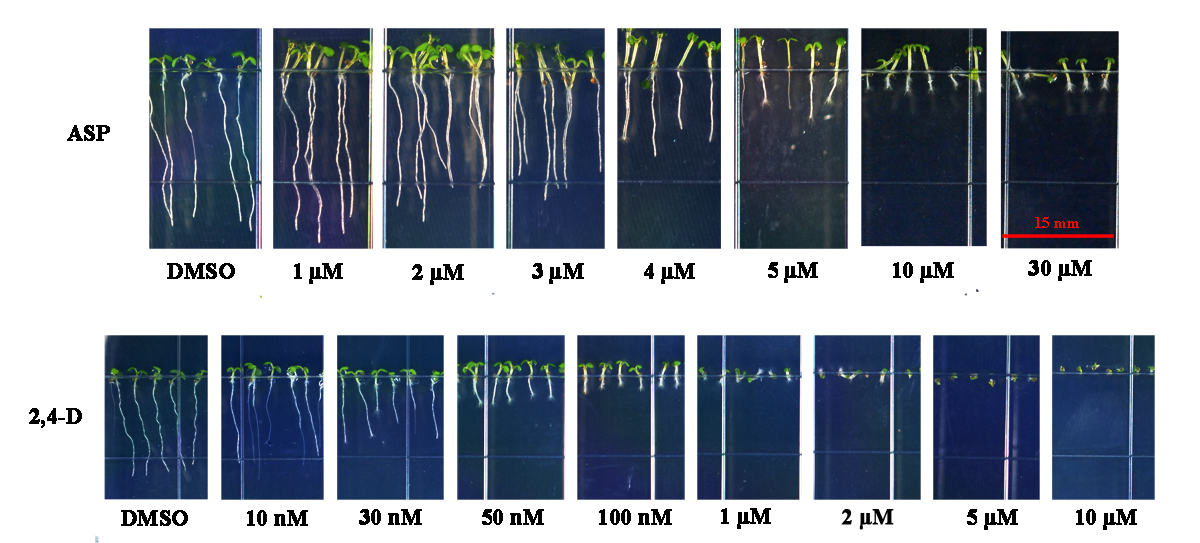

Supplement: Supplementary file 1 — Additional file 1: Fig. S1 Representative images of Arabidopsis WT (Col-0) seedlings grown on petri plates with medium supplemented with 2,4-D or ASP. DMSO was used as control. Concentrations are indicated under each image (Scale bar =15 mm). [file 12864_2020_7203_MOESM1_ESM.tif]

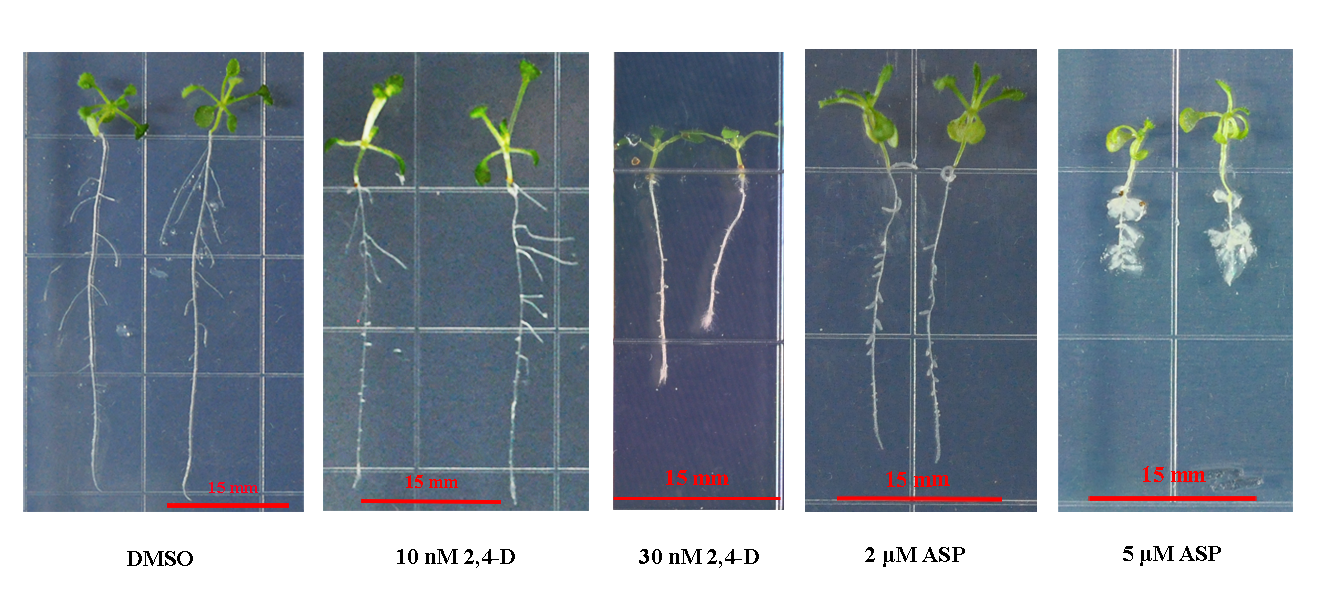

Supplement: Supplementary file 2 — Additional file 2: Fig. S2 Representative images of Arabidopsis WT (Col-0) seedlings supplemented with 2,4-D or ASP. Seedlings were grown on 1/2 MS liquid medium for nine days supplied with different concentrations of ASP or 2,4-D. DMSO was used as control. Concentrations are indicated under each image (Scale bar =15 mm). [file 12864_2020_7203_MOESM2_ESM.tif]

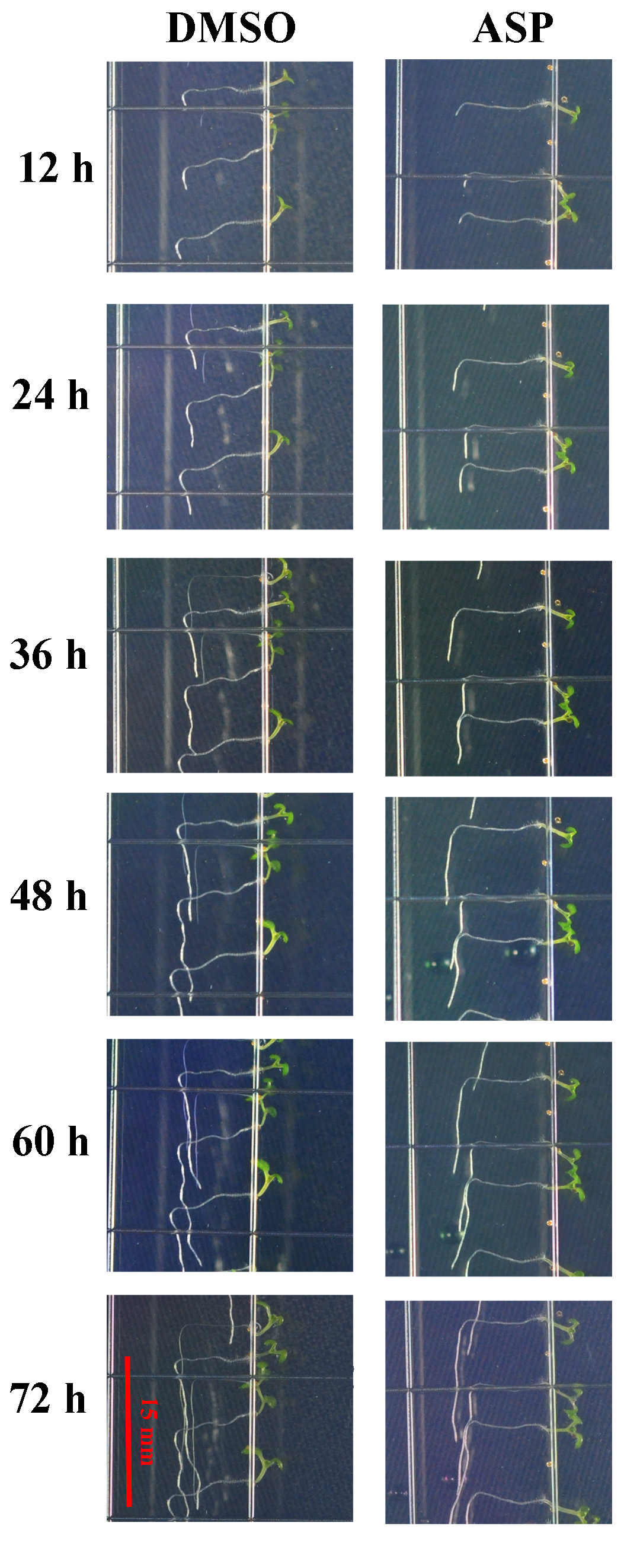

Supplement: Supplementary file 3 — Additional file 3: Fig. S3 Testing gravitropism of rotated roots with 2 μM ASP treatment for 12–72 h. [file 12864_2020_7203_MOESM3_ESM.tif]

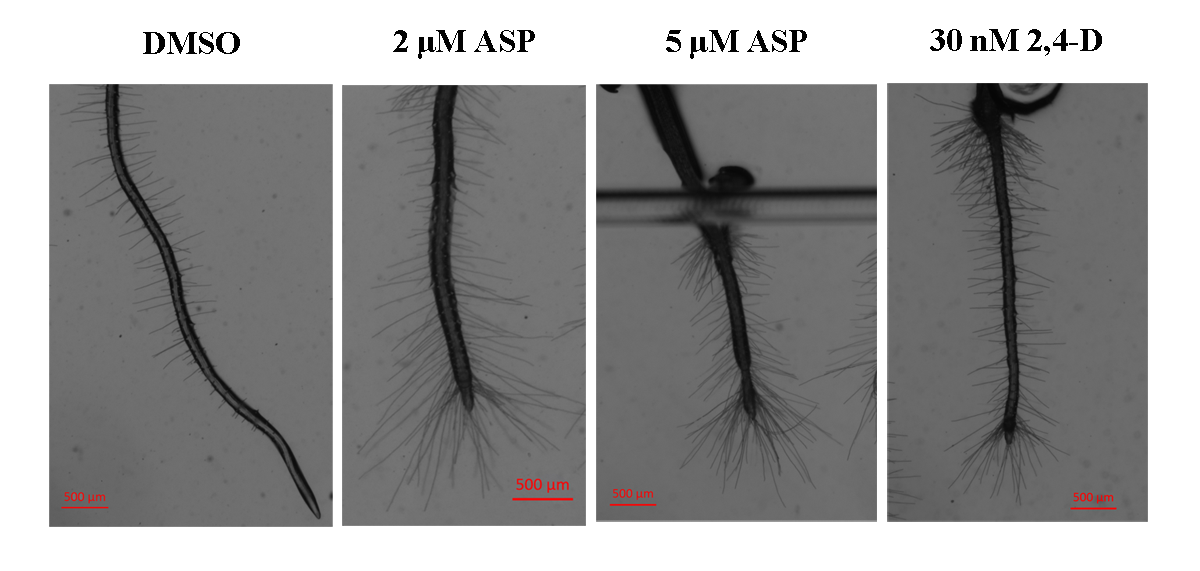

Supplement: Supplementary file 4 — Additional file 4: Fig. S4 Representative images showing the root-hair phenotypes of Arabidopsis WT (Col-0) seedlings. Seedlings were grown on ASP or 2,4-D-supplemented medium for 6 days. DMSO was used as control. Concentrations are indicated on each image (Scale bar = 500 μm). [file 12864_2020_7203_MOESM4_ESM.tif]

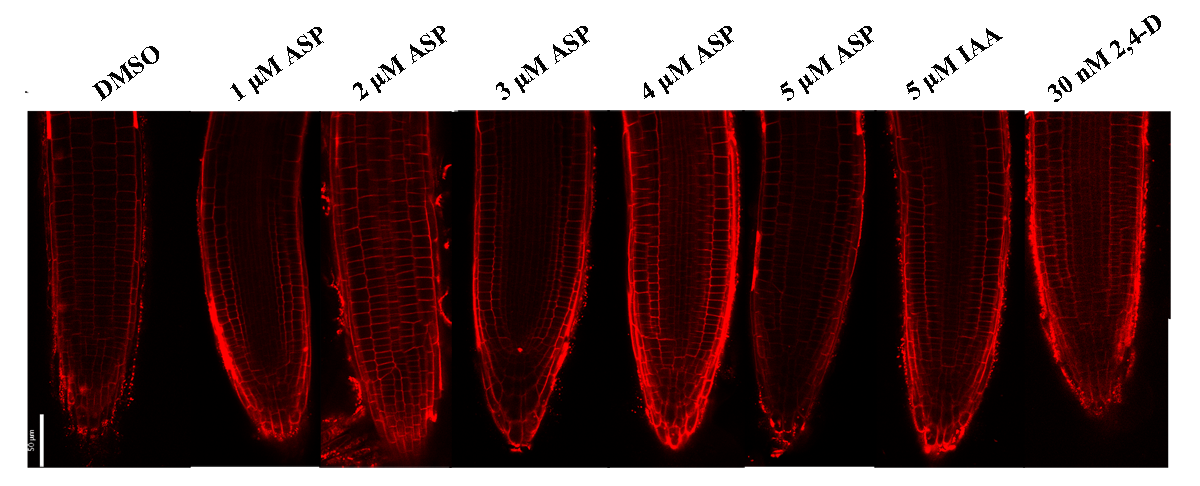

Supplement: Supplementary file 5 — Additional file 5: Fig. S5 Images of propidium iodide (PI)-stained root tip cells. Primary root tips from Col-0 seedlings, which grown on 1/2 MS medium with 0–5 μM ASP, 5 μM IAA and 30 nM 2,4-D for five days, stained with 10 mg/ml PI for 2 min. Images were acquired using a Zeiss LSCM 780 confocal microscope (Scale bar = 50 μm). [file 12864_2020_7203_MOESM5_ESM.tif]

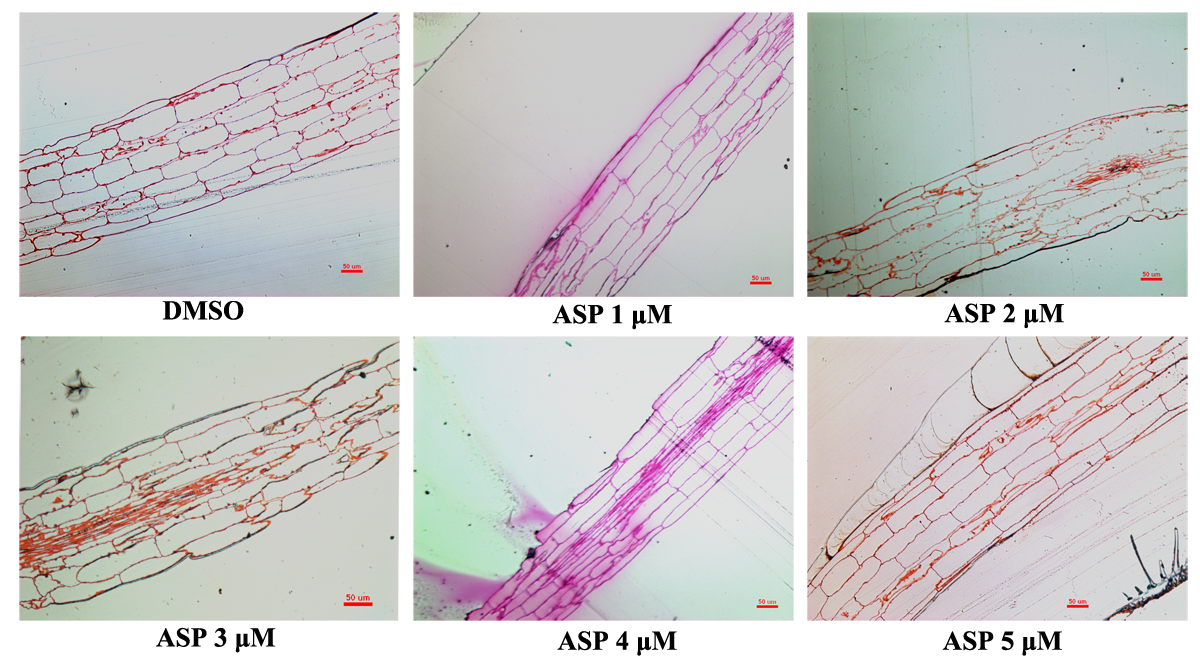

Supplement: Supplementary file 6 — Additional file 6: Fig. S6 ASP promoted hypocotyl cell elongation. Semi-thin longitudinal section cut from hypocotyl tissue cells and observed with light microscopy (Scale bar = 50 μm). [file 12864_2020_7203_MOESM6_ESM.tif]

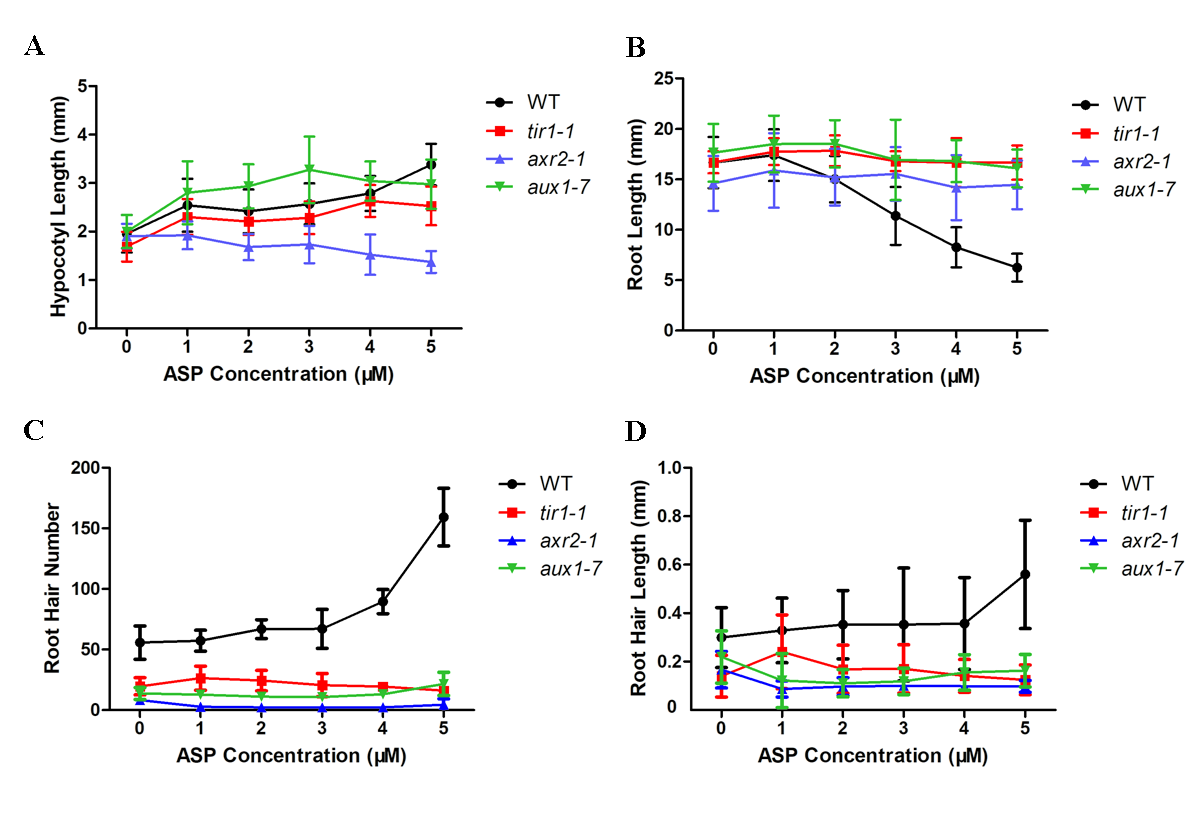

Supplement: Supplementary file 7 — Additional file 7: Fig. S7 Comparison of auxin signaling mutants (tir1–1, axr2–1, aux1–7) with wild-type seedlings on hypocotyl and root growth. The seedlings were grown on medium supplemented with 0–5 μM ASP for six days. A: Hypocotyl length. B: Root length. Means ± SD values were shown, N = 30 seedlings. C: Root hair number. D: Root hair length. Means ± SD values were shown, N = 10 seedlings. Micromolar concentrations are indicated on X axis. [file 12864_2020_7203_MOESM7_ESM.tif]

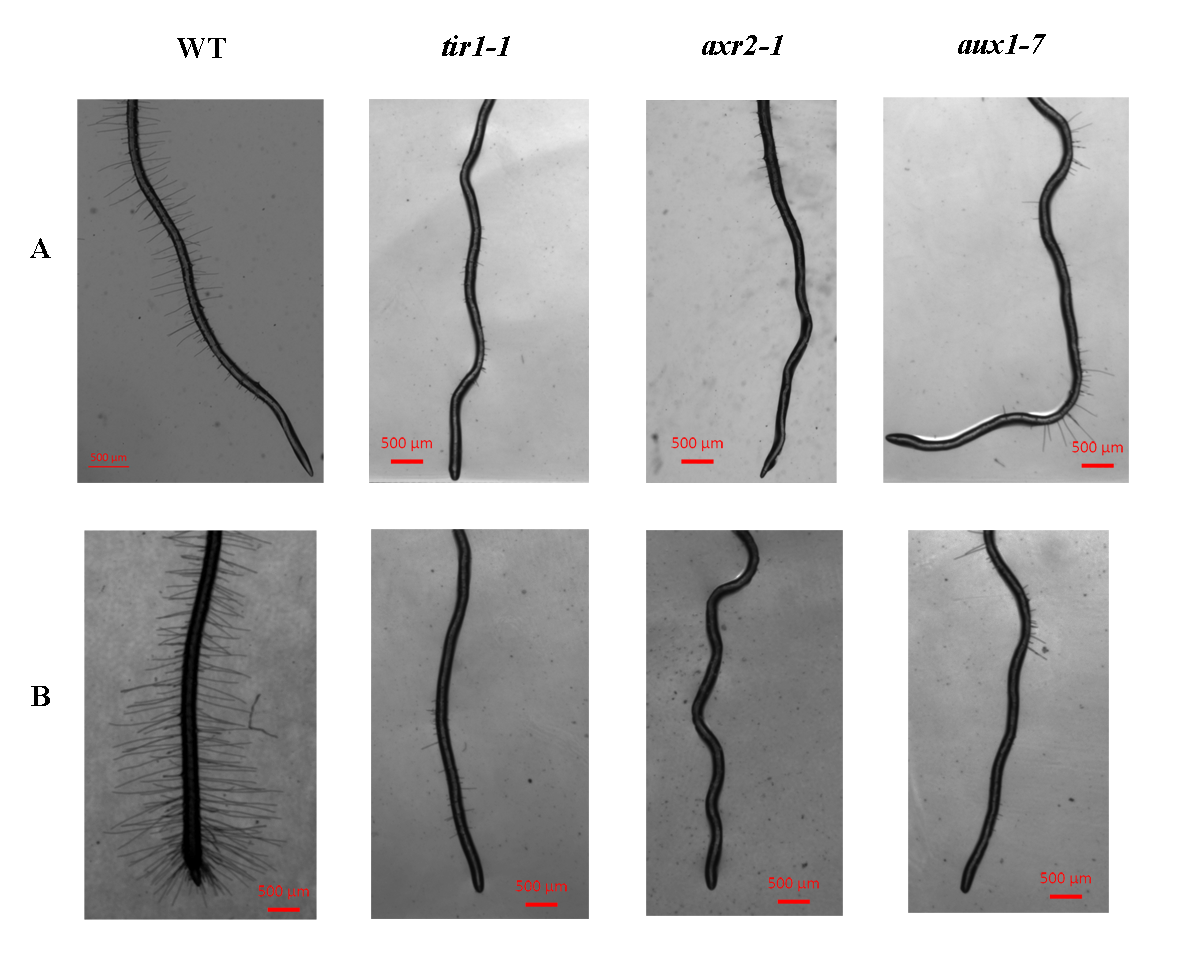

Supplement: Supplementary file 8 — Additional file 8: Fig. S8: Representative root hair phenotypes of auxin-related mutants. A: Wild-type and mutant (tir1–1, axr2–1, aux1–7) seedlings were grown on 1/2 MS liquid medium for 6 days. B: Wild-type and mutants were treated with 4 μM ASP for 6 days (Scale bar = 500 μm). [file 12864_2020_7203_MOESM8_ESM.tif]

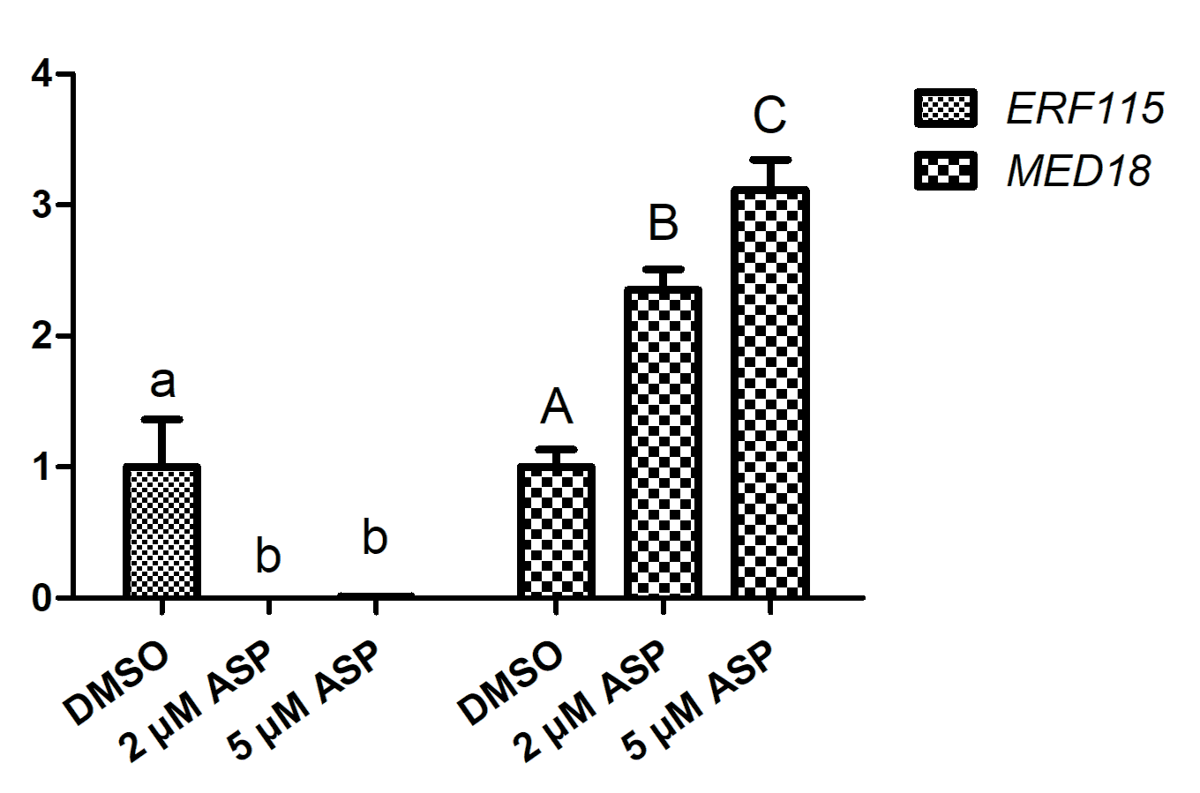

Supplement: Supplementary file 9 — Additional file 9: Fig. S9: RT-qPCR expression analyses of ERF115 and MED18 to test the root meristem cell viability. The seedlings were grown on 1/2 MS medium supplemented with 2 and 5 μM ASP for six days. DMSO was used as control. [file 12864_2020_7203_MOESM9_ESM.tif]
