# Supplementary material for: The piperazine compound ASP activates an auxin response in Arabidopsis thaliana
Source: BMC Genomics. 2020 Nov 11;21:788. doi: 10.1186/s12864-020-07203-8 (PMC7659159; doi:10.1186/s12864-020-07203-8)
Supplement: Supplementary file 10 — Additional file 10: Table S1 Sequences of primers used in RT-qPCR. [file 12864_2020_7203_MOESM10_ESM.docx]

The sequence of primers used in RT-qPCR

| **Primer Name** | **Forward primer** | **Reverse primer** |
| --- | --- | --- |
| *actin2* | TCTTCCGCTCTTTCTTTCCAAGC | ACCATTGTCACACACGATTGGTTG |
| *IAA2* | GAAGAATCTACACCTCCTACCAAAA | CACGTAGCTCACACTGTTGTTG |
| *GH3.5* | CCATCTCTGAGTTCCTCACAAGC | TCCTCTTCGATTGTTGGCATTAGC |
| *SAUR23* | TGGCTTTGGTGAGAAGTCTATT | TACTGAGCAGAGCTTGAAAAGA |
| *MED18* | GCTCCCGAGGACGATTTAGGAAG | ACCACCAACATGTTTAACCGTCCAT |
| *ERF115* | TCAAGTCATTGGAAACCAAAGC | ATAGTGCCTCTTCCTCAATAGC |
